# Phase-change VO_2_-based thermochromic smart windows

**DOI:** 10.1038/s41377-024-01560-9

**Published:** 2024-09-18

**Authors:** Cancheng Jiang, Lanyue He, Qingdong Xuan, Yuan Liao, Jian-Guo Dai, Dangyuan Lei

**Affiliations:** 1 Department of Materials Science and Engineering, Centre for Functional Photonics, and Hong Kong Branch of National Precious Metals Material Engineering Research Centre, City University of Hong Kong, Kowloon, Hong Kong 999077 China; 2 Department of Refrigeration and Cryogenics Engineering, Hefei University of Technology, 193 Tunxi Road, Hefei, 230009 China; 3 Department of Architecture and Civil Engineering, City University of Hong Kong, Kowloon, Hong Kong 999077 China

**Keywords:** Nanoparticles, Photonic devices

## Abstract

Thermochromic coatings hold promise in reducing building energy consumption by dynamically regulating the heat gain of windows, which are often regarded as less energy-efficient components, across different seasons. Vanadium dioxide (VO_2_) stands out as a versatile thermochromic material for smart windows owing to its reversible metal-to-insulator transition (MIT) alongside correlated structural and optical properties. In this review, we delve into recent advancements in the phase-change VO_2_-based thermochromic coatings for smart windows, spanning from the macroscopic crystal level to the microscopic structural level (including elemental doping and micro/nano-engineering), as well as advances in controllable fabrication. It is notable that hybridizing functional elements/materials (e.g., W, Mo/SiO_2_, TiN) with VO_2_ in delicate structural designs (e.g., core-shell, optical cavity) brings new degrees of freedom for controlling the thermochromic properties, including the MIT temperature, luminous transmittance, solar-energy modulation ability and building-relevant multi-functionality. Additionally, we provide an overview of alternative chromogenic materials that could potentially complement or surpass the intrinsic limitations of VO_2_. By examining the landscape of emerging materials, we aim to broaden the scope of possibilities for smart window technologies. We also offer insights into the current challenges and prospects of VO_2_-based thermochromic smart windows, presenting a roadmap for advancing this field towards enhanced energy efficiency and sustainable building design. In summary, this review innovatively categorizes doping strategies and corresponding effects of VO_2_, underscores their crucial NIR-energy modulation ability for smart windows, pioneers a theoretical analysis of inverse core-shell structures, prioritizes practical engineering strategies for solar modulation in VO_2_ films, and summarizes complementary chromogenic materials, thus ultimately advancing VO_2_-based smart window technologies with a fresh perspective.

## Abstract (web summary)

This review encapsulates the attributes of phase-change-VO_2_-based smart windows, highlighting their adaptive radiative cooling mechanisms, large modulation of both solar energy and thermal radiation, and methods for controlling phase-transition temperatures.

## Graphical abstract

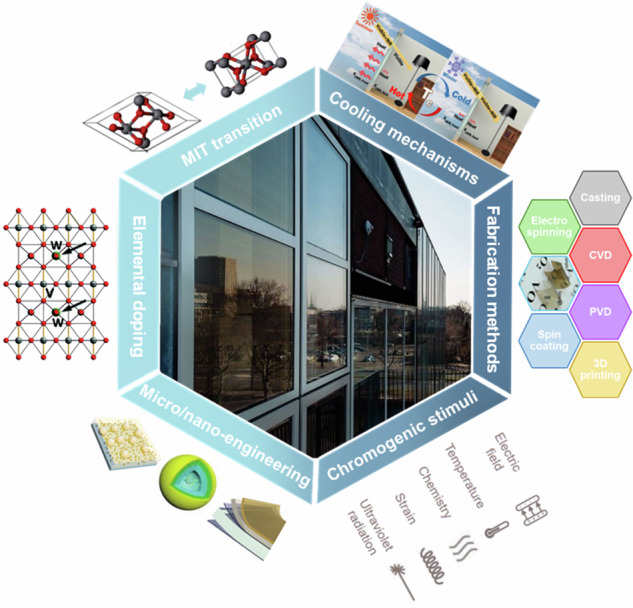

## Introduction

The escalating demand for energy consumption and the resultant emission of CO_2_ have been intensifying the global-warming problem, emphasizing the pressing imperative for energy conservation. Reports indicate that residential buildings are responsible for nearly 30% to 40% of the world’s primary energy consumption^1^. Hence, there is a significant imperative to enhance the energy efficiency of buildings. Among all building components, windows are frequently identified as less energy-efficient and requiring greater maintenance^2^, making smart windows an attractive research topic in optics, materials science, and building science. Windows play a crucial role in providing thermal, light, and acoustic comfort^3^, facilitating vision, air ventilation, photo-protection^4^, prevention of skin cancer^5^, and even influencing biopsychological effects^6^. The design of smart windows should take these aspects into consideration. Sunlight interacts with windows through transmission, reflection, or absorption, depending on their spectral properties in the ultraviolet (UV), visible, and near-infrared (NIR) regions. Consistently high transmittance in the visible spectrum is essential to meet lighting requirements. Indeed, a smart switch alternating between high and low transmittance in the UV and NIR spectral bands is desirable to minimize cooling loads during hot seasons and maximize heat gain during cold seasons. Thermochromic smart windows adjust radiation in invisible spectra according to surrounding temperatures, paving the way for highly efficient windows in the next generation of energy-conscious architectures.

Currently, commonly utilized thermochromic materials encompass vanadium dioxide, perovskite^7^, organic liquid crystal^8^, as well as mechano-thermo-chromic materials such as supersaturated salt hydrate crystal^9^. While supersaturated salt hydrates offer rapid and reversible phase transitions for smart window applications^9^, VO_2_ stands out for its sharp and abrupt change in transparency at a specific temperature, providing precise and consistent performance without the need for mechanical triggers or complex electro-thermal systems. VO_2_ is notable for the reversible temperature-dependent dielectric constants, exhibiting significant disparities between their metallic and insulating states. Rutile VO_2_(R) undergoes a reversible first-order MIT to monoclinic VO_2_(M) at a relatively moderate phase transition temperature (*T*_c_, 68 °C). The structure and optical properties can mutate before and after *T*_c_^10–13^, which makes VO_2_ an ideal thermochromic material for smart windows over the years.

**Fig. 1:**
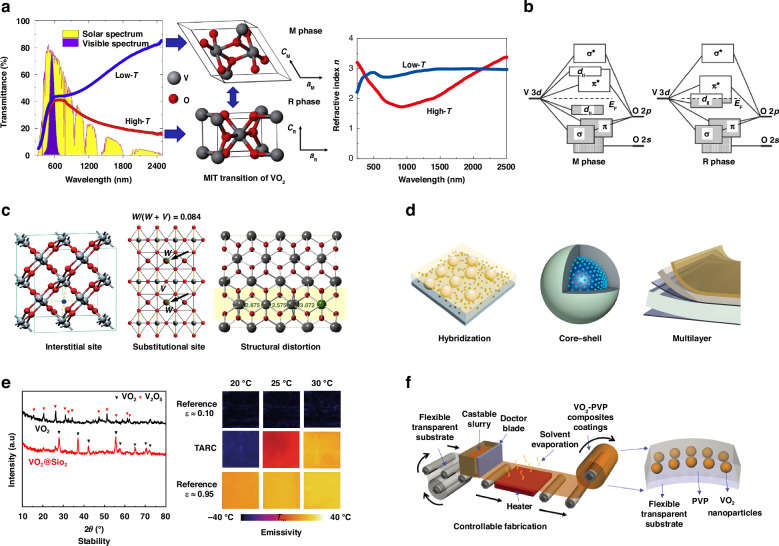
Overview of the VO_2_-based thermochromic coatings for smart windows. **a** Typical optical properties of VO_2_ thin films in visible and NIR regions and corresponding crystal structures: the monoclinic (M) insulating phase at low temperature and rutile (R) metallic phase at high temperature^168^. **b** Simplified band structures of VO_2_(M) and VO_2_(R) described by molecular orbital diagrams^169^. **c** The effect of elemental doping on the thermochromism of VO_2_^29,40,170^. **d** Micro/nano-engineering towards performance enhancement of VO_2_-based thermochromic coatings^48,70,171^. **e** Multifunctional VO_2_-based micro/nano-structures with stability^54^, emissivity^71^, etc. **f** Controllable fabrication methods of VO_2_-based thermochromic films/coatings^172^. Figures reproduced with permission from: (**b**), Whittaker et al.^169^, American Chemical Society

Here, we provide an overview of phase-change VO_2_-based thermochromic coatings for smart windows, drawing on the latest research and structured according to the logic depicted in Fig. 1. Firstly, the spectral transmittance modulation resulting from the MIT of VO_2_ in crystalline structures is introduced, as illustrated in Fig. 1a. Further exploration is undertaken into the effects of the MIT on the thermodynamic, electrical, and optical properties of VO_2_, elucidated through band structures depicted in Fig. 1b. Secondly, elemental doping is introduced as Fig. 1c illustrates, considering the difference between the actual temperature and *T*_c_. Thirdly, we discuss the micro/nano-engineering (Fig. 1d) in the categories including hybridization, core-shell micro/nanostructure, and multilayer films design, emphasizing on the structural effects for further improving the spectral modulation ability. Next, we review fabrication methods of VO_2_-based thermochromic films/coatings, with a specific focus on multi-functionalities, such as stability and emissivity, and the capability of controllable large-scale manufacturing, as depicted in Figs. 1e-1f. Additionally, we briefly introduce and discuss other promising chromogenic materials that can potentially be integrated with VO_2_ for smart windows to overcome their intrinsic limitations. Finally, outlooks and perspectives are addressed regarding current challenges and further advancements of VO_2_-based thermochromic smart windows.

This review distinguishes itself from previous works through the following unique contributions:

1. We provide a comprehensive categorization of VO_2_ doping strategies, shedding light on the doping mechanism, and emphasizing the crucial NIR-energy modulation capability (ΔT_NIR_) for smart window applications.
2. We pioneer a theoretical analysis of inverse core-shell structures featuring VO_2_ as the shell material, delving into their potential for enhancing solar modulation and offering novel insights for future research.
3. We prioritize practical engineering strategies to boost the solar-energy modulation capabilities of VO_2_ films, moving beyond mere descriptions of various VO_2_ nanostructures.
4. We offer a comprehensive summary of other chromogenic materials that can complement VO_2_ to enhance smart window functionality, providing a broader perspective than previous studies.

These elements inject a fresh perspective into VO_2_-based smart window technologies and are critical for further advancing the field.

## Temperature-triggered phase change for solar modulation

The MIT in VO_2_ is thermally induced, with concomitant alterations in the entropy and heat capacity of the material. In 1971, Dr. John B. Goodenough delineated molecular orbital diagrams grounded in crystal-field theory, as depicted in Fig. 1b^14^. Simply, there is a π bond and a π* anti-bond between the V^4+^ and O^2-^ orbitals in VO_2_(R). Meanwhile, a d_//_ nonbond is formed between nearby V^4+^ orbitals along the crystallographic c axis, which partially overlaps with the unfilled π* band. Metallic characteristics arise from the overlap between π* and d_//_ bonds, at which the Fermi level falls, leading to the VO_2_(R) with high electron mobility. As the temperature decreases, lattice distortion enhances the π overlap between the V^4+^ and O^2-^ orbitals, elevating the energy level of the π* anti-bond. Concurrently, the interaction between V-V pairs induces the splitting of the d_//_ bonds into d_//_-bonding and anti-bonding components, resulting in a band gap of ~0.7 eV between the π* and d_//_-bonding levels.

The presence of a band gap, resulting from the split between the π* and d//-bonding levels, gives rise to insulating properties in VO_2_(M). This band gap acts as a barrier to electron mobility, thereby leading to the manifestation of insulating behavior. While there may be variations between this theoretical proposal and experimental observations, it nonetheless offers valuable insights into the fundamental nature of the MIT in VO_2_. The fluctuation in electron mobility during the MIT enables switchable electrical conductivity, a characteristic that finds application in diverse fields such as tunable polarization converters^15,16^. In metallic VO_2_(R), the dielectric constant is considerably lower compared to insulating VO_2_(M). This discrepancy arises primarily because the presence of free electrons in VO_2_(R) allows them to screen the electric field effectively, diminishing the polarization of its lattice structure induced by external electric fields. As a result, the dielectric constant in metallic VO_2_(R) is notably reduced in comparison to its insulating counterpart. As depicted in Fig. 1a, VO_2_ exhibits a monoclinic structure characterized by high transparency to visible and near-infrared (NIR) light below its phase transition temperature (*T*_c_). Conversely, upon transitioning to the rutile structure (R) above Tc, the material maintains nearly consistent visible light transmittance but experiences a significant reduction in NIR transmittance^17–20^. To evaluate the optical performance of a VO_2_-based thermochromic smart window, the luminous transmittance (*T*_lum_, 380–780 nm), the solar-energy modulation ability (Δ*T*_sol_, 300–2500 nm) and the NIR-energy modulation ability (Δ*T*_NIR_, 780–2500 nm) are calculated as follow:

1 $${T}_{{\rm{lum}}/{\rm{sol}}/{\rm{NIR}}}=\frac{\int {\varPhi }_{{\rm{lum}}/{\rm{sol}}/{\rm{NIR}}}(\lambda )T(\lambda )d\lambda }{\int {\varPhi }_{{\rm{lum}}/{\rm{sol}}/{\rm{NIR}}}(\lambda )d\lambda }$$

2 $$\Delta {T}_{{\rm{sol}}}={T}_{{\rm{sol}},{\rm{c}}}-{T}_{{\rm{sol}},{\rm{h}}}$$

3 $$\Delta {T}_{{\rm{NIR}}}={T}_{{\rm{NIR}},{\rm{c}}}-{T}_{{\rm{NIR}},{\rm{h}}}$$

where *Φ*_lum_(*λ*) is the standard luminous efficiency function of human photosensitive vision^21–23^, *Φ*_sol/NIR_(*λ*) is the solar/NIR irradiance spectrum at air mass 1.5 (corresponding to the sun standing 37° above the horizon)^24^, *T*(*λ*) and *T*_lum/sol/NIR_ denote the spectral transmittance at wavelength *λ* and luminous/solar/NIR transmittance, respectively. The subscripts lum/sol/NIR and c/h represent the wavelength range (visible/whole solar spectrum/NIR) for integration and thermal states (low temperature/high temperature), respectively.

It’s noteworthy that *T*_lum_ differs from visible solar transmittance (*T*_vis_)^25^, which considers the relative spectral distribution of illuminant D65^26^. For VO_2_-based smart windows, Δ*T*_NIR_ is approximately double Δ*T*_sol_, as noticeable spectral transmittance changes occur in NIR bands (~52% of total solar irradiance) before and after MIT. Both high *T*_lum_ and Δ*T*_sol/NIR_ are crucial: the former signifies better lighting savings, while the latter determines the energy-saving performance of VO_2_-based smart windows. However, constrained by the intrinsic MIT, VO_2_-based smart windows face obstacles such as high transition temperature (68 °C), low luminous transmittance (*T*_lum_ < 60%), and weak solar-energy modulation (Δ*T*_sol_ < 15%). Extensive efforts have been dedicated to addressing these challenges and advancing VO_2_-based smart windows for practical applications.

## Elemental doping for tailoring the phase-transition temperature

As mentioned above, the primary challenge faced by VO_2_-based smart windows in practical applications is the significantly higher MIT compared to the normal operating temperature of conventional windows. To address this issue, researchers commonly employ elemental doping as the primary strategy to modulate *T*_c_ and optical properties of VO_2_. The effects of some doping elements are summarized in Table 1.

**Table 1:** Elemental doping effects on the thermochromic performance of VO_2_-based films

Doping strategies (chemical formula)		Dopant(s)	Doping level (at%)	*T*_lum_ (%)	Δ*T*_sol_ (%)	Δ*T*_NIR_ (%)	d*T*_c_/dx (°C/at%)	*T*_c_ (K)	Ref.
Carrier concentration increase	Interstitial site (VA_x_O_2_)	H	3	-	-	-	−38	227	^29,30^
		Li	3	-	-	-	−43	212	^31,32^
		Na	3	-	-	-	−49	293.15	^31,33^
		B	6	54.3	12.5	27.8	−63.3	301.25	^34,35^
	Substitutional site (V_1-x_A_x_O_2_/V_1-x-y_A_x_B_y_O_2_)	W	2	50.7	8	18.2	−20	301	^36,37^
		Mo	3	34.5	1.1	2.5	−9.33	313.01	^132,133^
		Nb	2.5	38.8	2.6	6.6	−8.4	320	^134,135^
		Sr + W	11.9 + 0.9	61.8	5.2	10	-	-	^136,137^
		Zr + W	4.2 + 2.1	60.7	10.6	22.7	−21.1	-	^138,139^
		Mo + W	7 + 8	55	-	-	−6.47 to 6.7	-	^140,141^
		Sn + W	1.9 + 1.6	41.1	13.4	25.7	-	-	^142,143^
Structural distortion (VA_x_O_2_/V_1-x_A_x_O_2_)		Sb	7	-	-	-	−11.7	259.1	^40,144^
		Be	3	-	-	-	−58	-	^41,145^
		Cr	3	22.4	7.4	19.1	0.69	343.07	^146,147^
		Ru	2.32	-	-	-	−10.5	316.64	^148,149^

“-” means data unavailable

The fundamental role of dopants is to lower the energy barrier, thereby reducing the MIT temperature, as the electronic phase transition in VO_2_ nearly coincides with the structural phase transition^27,28^. Correspondingly, the selection of the elemental doping is commonly based on two key factors: (1) Increasing the carrier concentration to accelerate the electronic phase transition: Dopants are selected to either donate additional electrons (n-type doping) or create spaces for electrons through the formation of vacancies (p-type doping). This tactic increases the mobile charge carrier density within the VO_2_ lattice, thereby amplifying the likelihood of electronic excitations that can precipitate the phase transition. By bolstering the number of available charge carriers, the dopants accelerate the electronic phase transition. This acceleration is due to the heightened probability of electronic interactions that can drive the system across the phase boundary, effectively diminishing the energy threshold for the MIT; (2) Introducing distortion into the atomic structure to assist the structural phase transition: The second prong of the doping strategy involves the deliberate introduction of atomic-scale distortions. These distortions arise from the mismatch between the dopants and the host VO_2_ lattice in terms of size or valence, leading to localized strain. Such strain can perturb the equilibrium of interatomic forces within the crystal, thus lowering the energy required for the structural reconfiguration that accompanies the MIT. The dopants act as a catalyst for structural changes by stabilizing the high-temperature metallic phase, which is characterized by a distinct arrangement of V-V dimers. This stabilization aids in the rapid reorganization of the vanadium and oxygen atoms, facilitating the transition from the insulating to the metallic state.

Regarding the increase in carrier concentration, researchers have pursued two primary doping strategies: a) insert smaller-sized doping atoms (Fig. 1c, left) such as H^29,30^, Li^31,32^, Na^31,33^, and B^34,35^ into the interstitial sites of VO_2_; b) substitute the V sites with high-valance elements (Fig. 1c, mid) such as W^36,37^, Mo^38^, Nb^39^. These strategies aim to enhance the concentration of mobile charge carriers within VO_2_ crystals, thus lowering *T*_c_. For instance, when smaller dopant atoms are inserted into the interstitial sites of VO_2_ lattice, they introduce additional electrons, thereby increasing the carrier concentration within VO_2_ crystals. Similarly, by substituting vanadium sites with high-valence elements, these elements can inject partial electrons into the valence band of VO_2_. For example, by substituting vanadium atoms in VO_2_, tungsten atoms can inject electrons into the valence band of VO_2_, effectively lowering *T*_c_, typically by 20–26 K/at%.

On the other hand, to address structural distortion, researchers have also employed methods such as adjusting the V-V distances in VO_2_ or modifying lattice parameters by a dopant (Sb^40^, Be^41^, etc.). Through these measures, researchers can effectively modulate the crystal structure of VO_2_, thereby affecting its transition temperature *T*_c_. For example, by adjusting the distances between vanadium atoms in VO_2_ crystals or by modifying lattice parameters, the structure of VO_2_ crystals can be altered to facilitate phase transition. These adjustments and distortions in structure can effectively reduce the transition temperature *T*_c_ of VO_2_, making it more suitable for applications such as smart windows.

In conclusion, elemental doping serves as an effective method for modulating the critical transition temperature of VO_2_-based smart windows, with selection based on precise control of carrier concentration and structural distortion. This strategy provides crucial technical support for enhancing the performance and applicability of VO_2_-based smart windows in practical applications. In future research, a deeper understanding of the doping mechanism and various influencing factors in the doping process will be essential.

While doping strategies have been successful in modulating the transition temperature (*T*_c_), the environmental stability and long-term performance impact of doped elements must be considered. Greater emphasis should be placed on the selection of dopants and the optimization of doping levels in future research to ensure more stable and efficient thermochromic smart windows.

## Micro/nano-engineering for Improving the solar-energy modulation ability

In addition to lowering *T*_c_, elevating *T*_lum_ and Δ*T*_sol_ is also important for smart window applications^42^. Micro/nano-engineering strategies through either micro- or macro-structural/materials modification have been proposed and investigated widely^43^. According to the modification principles, the reported micro/nano-engineering strategies can be classified into three categories: 1) hybridization that incorporates other materials/structures with individual VO_2_ materials, 2) core-shell micro/nano-structures that modulate the Mie/plasmonic resonant responses of spherical VO_2_ nanoparticles (NPs) as either core or shell, and 3) multilayer films that integrate other flat layers for enhanced optical resonances as well as multifunctionality.

### VO_2_-based hybridized thin films

**Fig. 2:**
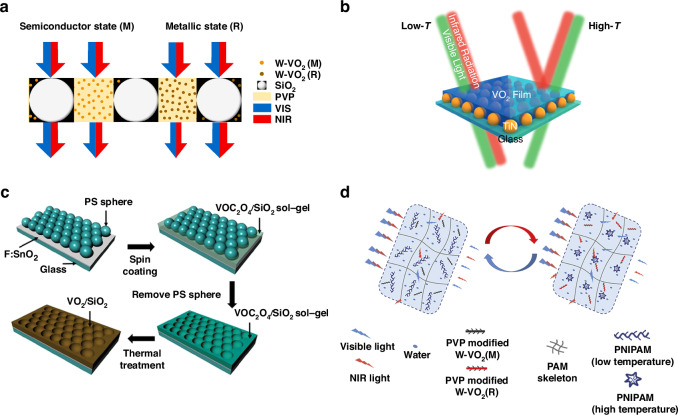
Thermochromic performance of VO_2_-based hybridized thin films. **a** Schematic diagrams of a W-VO_2_/SiO_2_/PVP composite film^48^. **b** Temperature-dependent optical properties of a VO_2_/TiN composite film^49^. **c** Schematics of fabrication process of a micro-structured VO_2_/SiO_2_ ordered composite film^50^. **d** Schematics of temperature-controlled structural changes of a W-VO_2_/PAM-PNIPAM hydrogel film^51^

In efforts to improve the thermochromic properties of VO_2_-based thin films, researchers often encounter a trade-off between Δ*T*_sol_ and *T*_lum_ when using thick layers of continuous VO_2_ film. To address this challenge, hybridization with other inorganic and/or organic materials has emerged as a viable strategy to optimize both *T*_lum_ and Δ*T*_sol_ simultaneously. This hybridization approach may affect the MIT and hysteresis-loop width of VO_2_ by inducing strains, while also modulating optical constants such as *T*_lum_ and color^44^. Furthermore, the introduction of functional materials like TiO_2_^45^ and ZrO_2_^46^ can add multifunctionality to the composite films, offering benefits such as self-cleaning and mechanical reinforcement. Researchers have explored various strategies to improve the optical performance of VO_2_-based thin films. Li et al. theoretically investigated the optical performance of VO_2_ NPs matrix composites and yielded much higher *T*_lum_ and Δ*T*_sol_ than pure VO_2_ thin films by calculations based on effective medium theory^47^. Liang et al. further introduced SiO_2_ microparticles in the W-doped VO_2_/PVP composites for *T*_lum_ improvement. The single layer of randomly dispersed SiO_2_ microspheres in the film provided optical pathways for sunlight, which offset the decrease of *T*_lum_ caused by the strong absorption of visible light by high volume concentration of W-VO_2_ NPs (Fig. 2a). Experimental results revealed that W-doped VO_2_/SiO_2_/PVP composite films enable high *T*_lum_ of 65% without sacrificing too much Δ*T*_sol_ (12.6%)^48^, which also proved the validity of the structure in the previous study by Li et al.^47^. Hao et al. proposed an alternative strategy that utilize plasmonic response to balance *T*_lum_ and Δ*T*_sol_. A plasmonic array of TiN NPs was fabricated underneath the VO_2_ thin film as Fig. 2b exhibits and endowed Δ*T*_sol_ of 10.8% and *T*_lum_ of 51%, respectively^49^. TiN NPs can efficiently absorb NIR radiation to provide local heating and accelerate the MIT in VO_2_. Additionally, the VO_2_ film can also be patterned for performance improvement. Cao et al. synthesized periodical and spherical cavity-structured VO_2_/SiO_2_ composite films through a polystyrene template and annealing process as illustrated in Fig. 2c. Under the photonics crystal structure of the patterned VO_2_ composite film, balanced thermochromic properties can be obtained as Δ*T*_sol_ of 8.4% and *T*_lum_ of 55.6%^50^. Inorganic VO_2_ could also be integrated with organic materials to enhance the thermochromic properties. For instance, He et al. presented a dynamically regulated system based on W-VO_2_/PAM-PNIPAM hydrogel films, which was developed into smart windows (Δ*T*_sol_ 46.3% and *T*_lum_ 72%)^51^. As illustrated in Fig. 2d, the PVP-modified monoclinic W-VO_2_ was dispersed in the PNIPAM microgel, where the PAM hydrogel with high *T*_lum_ serves as a skeleton, holding and keeping water molecules, PNIPAM microgel and surface-modified W-VO_2_ inside. Upon heating, the intramolecular and intermolecular hydrophobicity of PNIPAM is enhanced, leading to the discharge of water molecules from the chemical framework of PNIPAM and generating phase separation interfaces. These interfaces can strongly scatter the incident solar irradiance when the W-VO_2_ undergoes the MIT and hence dramatically enhance the NIR light reflectivity simultaneously.

### VO_2_-based core-shell micro/nano-structures

**Fig. 3:**
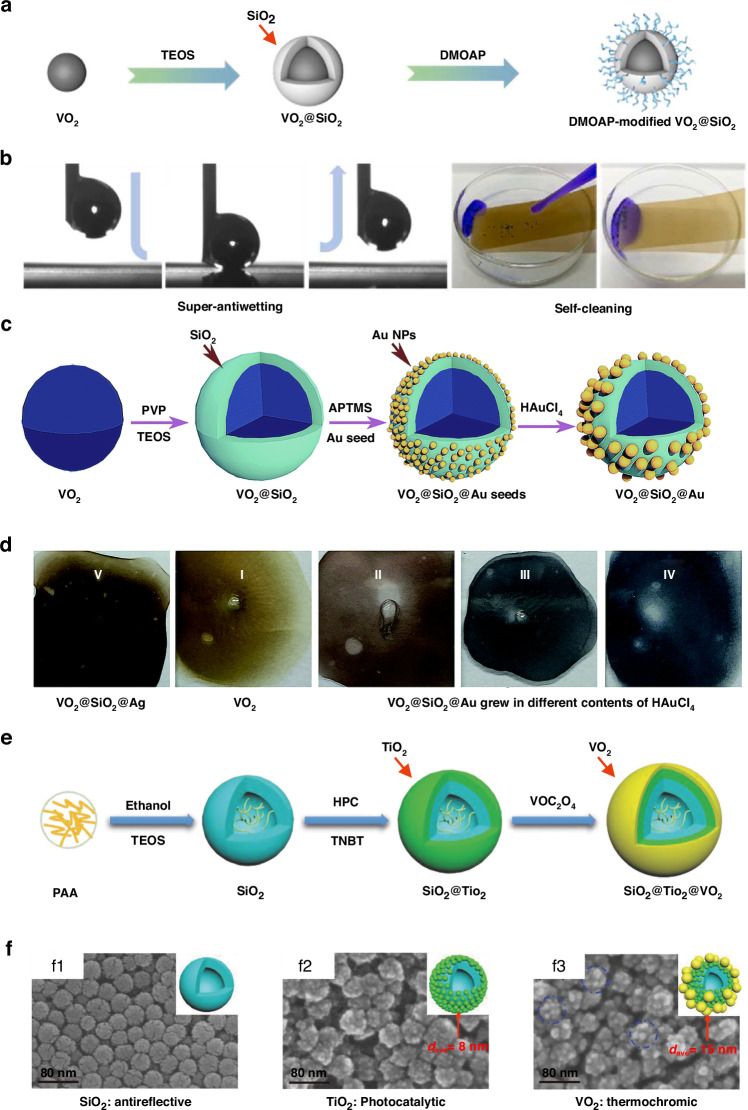
Thermochromic performance of VO_2_-based core-shell micro/nanostructures. **a** Synthesis procedures of VSQ core-shell NPs^54^. **b** Low water adhesion and self-cleaning performance of a VSQ-coated surface^54^. **c** Synthesis procedures of VO_2_@SiO_2_@Au trilayer core-shell NPs^56^. **d** Different colors of VO_2_-based trilayer core-shell NPs-based films. From left to right: a VO_2_@SiO_2_@Ag NPs-based film (**V**), a plain VO_2_ film (**I**), VO_2_@SiO_2_@Au films grew in different content of HAuCl_4_ (**II-IV**)^56^. **e** Synthesis procedures of SiO_2_@TiO_2_@VO_2_ inverted trilayer core-shell NPs^57^. **f** SEM micrographs of (**f1**) a SiO_2_ hollow nanosphere-based coating, (**f2**) a SiO_2_@TiO_2_ coating, and (**f3**) a SiO_2_@TiO_2_@VO_2_ coating, respectively^57^

In addition to hybridization methods, core-shell approaches have emerged as an efficacious strategy for enhancing the thermochromic properties and functionalities of VO_2_-based films^52^. These core-shell nanostructures can be broadly categorized into bilayer and trilayer configurations based on the number of layers, wherein VO_2_ is encapsulated within dielectric shells such as SiO_2_, TiO_2_, SnO_2_, ZnO, Al_2_O_3_^53^, or metallic shells like Au and Ag. For instance, Pi et al. prepared thermochromic and hydrophobic core-shell VO_2_@SiO_2_ (VSQ) NPs with tetraethoxysilane (TEOS) and dimethyloctadecyl [3-(trimethoxysilyl) propyl] ammonium chloride (DMOAP) as depicted in Fig. 3a^54^. The Δ*T*_sol_ reaches up to 15.4% while the *T*_lum_ remains as high as 51.5% owing to localized surface plasmon resonance (LSPR)^55^. Furthermore, the SiO_2_ shell efficiently shields VO_2_ from oxidation, enhancing its durability, while DMOAP modification imparts additional functionalities including anti-agglomeration, surface hydrophobicity, and self-cleaning, as illustrated in Fig. 3b^54^. Lu et al. synthesized VO_2_@SiO_2_@Au trilayer core-shell nanoparticles to harness LSPR and enhance optical tunability, as depicted in Fig. 3c^56^. By modulating the material composition and particle sizes of the outer metallic shell, the resonance peak could be tuned across a wide spectral range, offering customizable colorful appearances for the films, as shown in Fig. 3d. Since VO_2_ can transition between dielectric and metallic states, it can be employed as either the inner or outer layer of core-shell structures. Yao et al. fabricated SiO_2_@TiO_2_@VO_2_ inverted trilayer core-shell NPs via controlled interfacial engineering, as depicted in Fig. 3e. The *T*_lum_ of the resultant coating reached a remarkable 74%. Additionally, as Fig. 3f illustrates, since TiO_2_ was partially exposed rather than completely covered by VO_2_, the coating facilitated the integration of thermochromism from the outer VO_2_ layer, photocatalytic self-cleaning capability from the middle TiO_2_ layer, and antireflective properties from the internal SiO_2_ hollow nanospheres^57^.

**Fig. 4:**
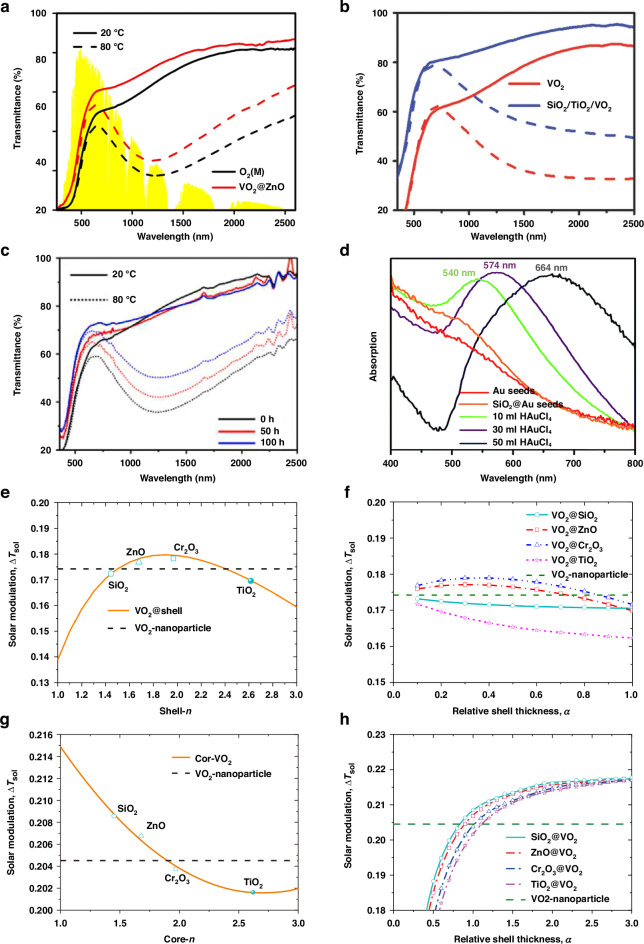
Optical and thermal performance of VO_2_-based films and coatings. **a–c** Transmittance spectra of **a** VO_2_ and VO_2_@ZnO films at 20 and 80 °C^173^, **b** VO_2_ and SiO_2_@TiO_2_@VO_2_ coatings at 20 and 100 °C^57^, and **c** VSQ coatings treated at 60 °C and 90% RH for different periods^54^. **d** Absorption spectra of different solutions^56^. **e, f** Δ*T*_sol_ of VO_2_ core-shell NPs-based films, as a function of **e** shell refractive index and **f** relative shell thickness^53^. **g, h** Δ*T*_sol_ of VO_2_ inverted core-shell NPs-based films, as a function of **g** core refractive index and **h** relative shell thickness, the performance of the plain VO_2_ film was shown by the dashed lines. Figures reproduced with permission from: **a**, Chen et al.^58^, American Chemical Society

The spectral transmittances of typical bilayer and trilayer VO_2_-based core-shell structures are shown in Fig. 4a, b, demonstrating a notable enhancement in *T*_lum_. Additionally, Fig. 4c, d illustrates the durability and coloration of these core-shell configurations, providing detailed insights into their multifunctional capabilities. Specifically, Fig. 4c showcases the VSQ coating’s ability to enhance the stability and longevity of VO_2_ by providing a protective SiO_2_ shell, preventing oxidation, incorporating hydrophobic modification for self-cleaning, and maintaining structural integrity through robust adhesion. This innovative approach collectively improves environmental and mechanical durability, addressing crucial challenges faced by VO_2_-based thermochromic coatings^55^. Moreover, in Fig. 4d, the tunable SPR properties of Au nanoparticles are highlighted, demonstrating their potential to modify the color of solutions and, by extension, the color of VO_2_-based thermochromic smart films^57^. Lu et al.‘s work illustrates how controlling the size of the Au nanoparticles enables the tailoring of film color from brick red to purple and blue. This advancement represents a significant stride in the development of smart window technologies, offering versatile solutions for enhancing the aesthetic appeal and functionality of VO_2_-based coatings. The thermochromic behavior of VO_2_-based core-shell structures is intricately linked to the properties of the core/shell materials and their respective sizes, which govern the dielectric environment surrounding VO_2_^54^. To systematically investigate the optical performance of the thermochromic films, Xie et al. performed theoretical calculations based on the effective medium theory coupled with the transfer matrix method. Their findings, showcased in Fig. 4e–f, suggest that for films composed of VO_2_ core-shell nanoparticles, selecting a low absorption shell material (e.g., ZnO or Cr_2_O_3_) with a refractive index (RI) ranging from 1.6 to 2.3, and maintaining a relative shell thickness between 0.1 and 0.3, is crucial for preserving a high sol-gel transition temperature difference (Δ*T*_sol_)^53^. Here, the relative shell thickness (α) is defined as the ratio of the shell thickness to the core radius (α = *t*_shell_ /*R*_core_). In Fig. 4e, dashed lines represent the solar modulation capability of VO_2_ nanoparticle, while solid orange lines represent VO_2_@shell structures with varying shell materials. Analysis indicates that VO_2_-based core-shell structures outperform VO_2_ nanoparticles when the shell refractive index falls within 1.6 to 2.3, yielding performance enhancement. Similarly, in Fig. 4f, for VO_2_@ZnO or VO_2_@Cr_2_O_3_ structures, superior performance of VO_2_-based core-shell structures is observed with shell thickness between 0.1 and 0.3, demonstrating performance improvement. Table 2 has been included, detailing the distinct performance and characteristics of VO_2_-based core-shell micro/nanostructures with various dielectric and metallic shell materials.

**Table 2:** Performance comparison of different VO_2_ core-shell structures

No.	Core Material	Shell Material	*T*_lum_ (%)	Δ*T*_sol_ (%)	Characteristics/Performance	Ref.
1	VO_2_	SiO_2_	51.5	15.4	High weatherability, high Δ*T*_sol_ and *T*_lum_	^55^
2	VO_2_	SiO_2_@ Au	-	7.52	Trilayer core-shell structure, high optical tunability	^57^
3	SiO_2_	Au@ VO_2_	74	12	Inverted trilayer core-shell structure, high thermochromism benefiting from partially exposed TiO_2_	^58^
4	VO_2_	ZnO	-	-	Low absorption shell material, high Δ*T*_sol_	^54^
5	VO_2_	Cr_2_O_3_	-	-	Low absorption shell material, high Δ*T*_sol_	^54^
6	VO_2_	SiO_2_	51.5	15.4	Metallic shell, high LSPR effect	^56^
7	VO_2_	Ag	-	-	Metallic shell, high LSPR effect	^56^

“-” means data unavailable

However, the scarcity of investigations into the optical characteristics of inverted core-shell structures, where VO_2_ serves as the shell material, underscores a critical gap in our current understanding. To address this gap, we conducted theoretical analyses utilizing the Mie theory coupled with the Monte Carlo method. Our research specifically targeted inverted VO_2_ core-shell nanoparticles incorporating core materials such as SiO_2_, ZnO, Cr_2_O_3_, and TiO_2_.

Upon scrutinizing Fig. 4e, g, a notable trend emerged: the inverted structures consistently displayed superior solar modulation capability across all material combinations compared to their conventional counterparts. Furthermore, it was observed that the Δ*T*_sol_ of these films could be further enhanced by integrating low-absorption core materials, like SiO_2_, characterized by a low refractive index and a relative shell thickness exceeding 1.1, as shown in Fig. 4g, h. These results reveal that the inversion of the core-shell structure leads to enhancements in Mie scattering effects, suggesting that inverted structures hold greater potential for solar modulation capabilities compared to ordinary structures.

### Multilayer films design with photonic cavity

In general, there are mainly two forms of thermochromic coatings based on VO_2_: flexible foils and multilayered films. Flexible foils involve dispersing VO_2_-based nanoparticles in a polymer host matrix such as PU, PVP, and PMMA, and then combining them with a flexible substrate. On the other hand, multilayered films are typically fabricated on glass using direct deposition methods such as physical vapor deposition (PVD) and chemical vapor deposition (CVD). Since the absorption of VO_2_ is mainly in the 250–700 nm wavelength range, the *T*_lum_ of single-layer VO_2_ films is generally restricted. Additionally, due to its transmittance switches primarily in the NIR region, which accounts for about 43% of solar energy in the solar spectrum, leading to limited solar modulation (Δ*T*_sol_), often remaining below 10%. Multilayer films design is an effective solution towards this perplexity. To address these limitations, multilayer film designs have emerged as effective solutions. These films not only allow for the design of solar transmittance but also enable the tailoring of NIR emissivity^58^. Moreover, the integration of different layers makes it easier to achieve multifunctionality compared to single-layer films.

**Fig. 5:**
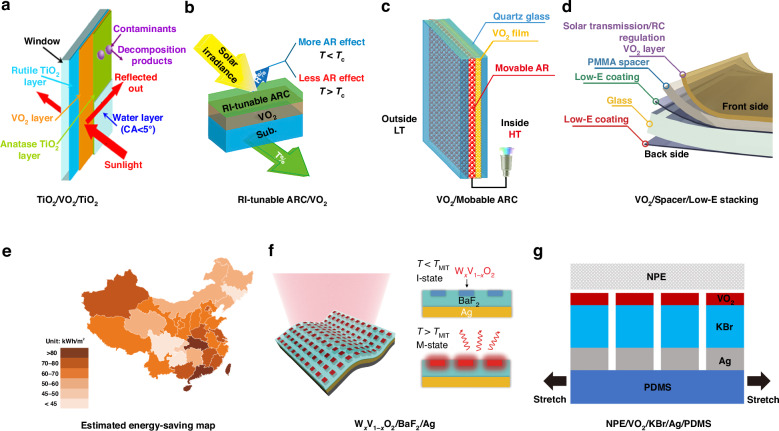
Multilayer design to enhance the thermochromism and multifunctionality of VO_2_-based thin films. **a** Schematic diagrams of a TiO_2_/VO_2_/TiO_2_ double-layer AR design^63^. **b** A RI-tunable ARC/VO_2_ bilayer^64^. **c** A VO_2_/movable ARC bilayer sandwiched between two quartz glass^65^. **d** A multilayer system as a RCRT window^70^. **e** Estimated energy-saving map of multilayer VO_2_-based thermochromic smart window. **f** A tunable Fabry-Pérot cavity based TARC design^71^. **g** A VO_2_-PDMS-driven radiative cooling coating^77^. Figures reproduced with permission from: (**c**), Xu et al.^66^, American Chemical Society

Antireflection (AR) is a noteworthy function that has been demonstrated as a viable method to enhance the low luminous transmittance (*T*_lum_) of VO_2_-based films without compromising their thermochromic properties. Additionally, neat VO_2_ is prone to oxidation to V_2_O_5_ in air and exhibits poor acid resistance, leading to environmental instability that limits its practical application as a thermochromic coating in smart windows^59^. Incorporating VO_2_ films with other layers can introduce practical functions such as antioxidizability^60^, hydrophobicity^61^, and photocatalysis^62^. For instance, Zheng et al. designed a TiO_2_/VO_2_/TiO_2_ multilayer film (with Δ*T*_sol_ of 10.2% and *T*_lum_ of 30.1%), which exhibited at least three functions: antifogging/self-cleaning, thermochromic, and antireflective properties. These properties were respectively attributed to the top TiO_2_(A), the middle VO_2_(M), and the bottom TiO_2_(R) layers, as illustrated in Fig. 5a^63^. However, as for the antireflection coatings (ARCs), most researchers focused on increasing luminous transmittance (*T*_lum_) at the expense of sacrificing solar modulation (Δ*T*_sol_). This trade-off is a significant challenge as Δ*T*_sol_ is a determinant factor for energy-saving applications and can hinder the translation of these technologies from the laboratory to the market. To address this challenge, variable ARC/VO_2_ multilayer film designs, as illustrated in Fig. 5b and c, have emerged. For example, Liu et al. utilized a novel RI-tunable (1.47–1.92 at λ = 550 nm) nano sol-gel-based ARC/VO_2_ bilayer (Fig. 4b) with a Δ*T*_sol_ of 18.9% and *T*_lum_ of 44% to enhance the antireflective effect at lower temperatures. This approach aims to maximize Δ*T*_sol_ for various VO_2_ nanosubstrates^64^. Xu et al. fabricated a VO_2_/Movable ARC bilayer controlled by dual modes using magnetron sputtering, as illustrated in Fig. 5c. This approach significantly improved the thermochromic properties, with a Δ*T*_sol_ of 18.2% and *T*_lum_ of 42.5%. Moreover, it enabled the two-phase smart regulation of eco-friendly H_2_O through the conversion of liquid and gaseous states. This bilayer system can passively modulate according to the variation in temperature difference between indoor and outdoor environments. Additionally, it can actively control the amount of solvent by detecting the temperature difference and humidity between the insulating glass, thereby adjusting the thickness of the ARC^65^.

Thermal emissivity (${\varepsilon }_{{\rm{T}}}$) holds equal importance alongside Δ*T*_sol_ and *T*_lum_ for VO_2_-based smart windows. It has been leveraged in numerous thermal management and energy-saving applications^66–69^ and has recently garnered attention in the context of smart windows, particularly in combination with the concept of radiative cooling^70–72^. Radiative cooling harnesses the infrared atmospheric transparent window to dissipate heat and effectively cools terrestrial objects through thermal radiation^73–75^.

In comparison to traditional smart windows, which primarily focus on modulating incoming solar energy, high thermal emissivity (${\varepsilon }_{{\rm{T}}}$) for radiative cooling offers an additional channel for dissipating excessive thermal energy generated by solar heating. According to Kirchhoff’s thermal law, the absorptivity of an object is equal to its emissivity in thermal equilibrium conditions. And the transmittance of glasses can be ignored when the wavelength is longer than 4.5 μm. Commonly, ${\varepsilon }_{{\rm{T}}}$ is calculated by weighting the film reflectance with the black-body emission spectrum from 4.5 to 25 µm as follow:

4 $${\varepsilon }_{{\rm{T}}}=\mathop{\sum }\limits_{4.5}^{25}{G}_{{\rm{T}}}\left(\lambda \right)E\left(\lambda \right)\Delta \lambda \approx 1-\mathop{\sum }\limits_{4.5}^{25}{G}_{{\rm{T}}}\left(\lambda \right)R\left(\lambda \right)\Delta \lambda$$

where *G*_T_(*λ*) is the normalized relative spectral distribution of black-body radiation at temperature *T* (*T* is chosen to be 20 °C according to CNS GB/T 1895.2–2002). *E*(*λ*) refers to the spectral emittance, i.e., the fraction of the black-body radiation. *R*(*λ*) refers to the reflectance in the region (4.5-25 µm).

A high value of ${\varepsilon }_{{\rm{T}}}$ indicates an intensive energy exchange between the window surface and its ambience through thermal radiation and absorption^76^. Wang et al. developed a passive radiative cooling regulating thermochromic (RCRT) smart window (Δ*T*_sol_ 17% and *T*_lum_ 32.2%) with tunable long-wave infrared (LWIR) emissivity (${\varepsilon }_{{\rm{LWIR}}}$) based on a W-doped VO_2_-PMMA/spacer/low-E stack using spin coating, as illustrated in Fig. 5d. This configuration formed a Fabry-Pérot resonator, enabling the promotion of radiative cooling during warm weather (high ${\varepsilon }_{{\rm{LWIR}}}$) or suppress it during cold weather (low ${\varepsilon }_{{\rm{LWIR}}}$)^70^.

To further elucidate the energy-saving benefits of VO_2_-based smart windows, we calculated the annual total energy savings for model buildings equipped with multilayer VO_2_-based smart windows (Δ*T*_sol_ = 10%, Δ*ε* = 0.5). Figure 5e illustrates the total energy saved annually in various cities across China. From the energy-saving map, it is evident that cities in southern regions exhibit more pronounced energy-saving effects compared to those in northern regions, primarily due to the hotter climate. For instance, the average annual energy savings in Hong Kong amount to 80.16 kWh/m^2^, whereas in Jilin, it is only 45.12 kWh/m^2^. This analysis underscores that the installation of VO_2_-based smart windows in buildings leads to reduced energy consumption, yielding significant energy-saving benefits.

It is worth noting that achieving dynamic modulation of emissivity can be challenging when incorporating low-E coatings (such as ITO) due to their strong NIR blocking properties, which can lead to relatively low values of Δ*T*_sol_. The simultaneous modulation of the spectral performance in both the NIR and long-wave infrared (LWIR) regions remains a question to be addressed in future research.

Additionally, Tang et al. introduced a W_x_V_1-x_O_2_-based temperature-adaptive radiative coating (TARC), as shown in Fig. 5e, to achieve strong sky-window radiative cooling at high temperatures and solar heating or warm-keeping at low temperatures. This was made possible by amplified radiative absorption facilitated by the designed Fabry-Pérot resonance with adjacent W_x_V_1-x_O_2_ blocks, as well as the bottom Ag layer within the ¼-wavelength cavity^71^. Furthermore, Liu et al. presented a VO_2_-PDMS-driven intelligent radiative cooling coating with both automatic cooling switching and continuous adjustment of thermal comfort, as illustrated in Fig. 5f^77^. The polydimethylsiloxane (PDMS) substrate could be stretched to modulate the radiative cooling power according to our desired cooling temperature. Adjusting the VO_2_-PDMS coatings through stretching modifies the filling ratio, consequently influencing the dielectric characteristics of the stacked nano-grating structure. This alteration impacts surface waves at the interfaces, resulting in modifications to the spectral performance of the coatings.

Micro/nano-engineering, offering new degrees of freedom in the design and manufacturing of VO_2_-based thermochromic materials, also presents challenges regarding manufacturing complexity and cost control. It is suggested that future efforts should be directed towards streamlining the manufacturing process and reducing costs, without compromising or enhancing material performance.

To develop high-performance and cost-effective VO_2_-based smart windows, researchers must gain a deep understanding and precise control of the doping mechanisms, utilize micro/nano-engineering techniques to optimize optical properties, and balance *T*_c_ with optical characteristics. By employing precise doping strategies and micro/nano-engineering techniques, *T*_lum_ and Δ*T*_sol_ can be optimized. Additionally, simplifying the manufacturing process to reduce costs is essential to ensure the product’s performance and economic viability in practical applications.

## Methods

### Preparation of VO_2_ nanoparticles

High-quality VO_2_ NPs with narrow distribution and uniform morphology are undoubtedly conducive to fabricate ideal thermochromic films. Recently, several methods have been reported to prepare definitive VO_2_ particles, as shown in Table 3. For instance, solid-phase reaction methods offer low-cost and large-scale production but may involve toxic reactants and impurities, while gas-phase reaction methods provide morphological control but are often constrained by conditional reactions and equipment complexity.

**Table 3:** Summary of the preparation methods of VO_2_ particles

Reaction environment	Method	Pros	Cons	Particle size	Particle shape	Ref.
Solid phase reaction	Thermal reduction	Low-cost, large-scale	Toxicity of reactant, conditional reaction, impurities	Micro	Rhombohedral	^150,151^
	Pyrolysis	Mild reaction, controllable composition, less impurities	CO_2_ emission, large particle size	Nano	Rod	^152,153^
	Ball-milling	Large-scale, modifiable	Introducing impurities	Nano	Irregular	^154,155^
Gas phase reaction	Physical vapor deposition	Morphological control	Conditional reaction	Micro/nano	Rod	^156,157^
	Chemical vapor deposition	High crystallinity, uniform	Small-scale, substrate-dependent	Nano	Wire	^158,159^
Liquid phase reaction	Sol-gel method	Low-cost, facile	Shrinkage upon drying	Nano	Spherical	^160,161^
	Hydrothermal method	Low-cost, high crystallinity	Conditional reaction	Nano	Snowflake	^162,163^
	Seeded growth	Controllable crystal growth	Pressure requirement	Nano	Star	^164,165^
	Solution combustion	Low-cost, time-saving, controllable composition	Small-scale, uneven grainsize	Micro/nano	Irregular	^166,167^

CVD and PVD methods are typically constrained by expensive and intricate equipment, often resulting in low yield. The hydrothermal method usually necessitates precise control over temperature and atmosphere, while ball-milling and thermal reduction methods are prone to introducing impurities. In contrast, the pyrolysis process offers advantages such as mild reaction conditions, controllable product composition, and minimal impurity introduction, making it conducive to large-scale production. Recent advancements, such as the combination of solvent-thermal and pyrolysis processes, have led to the synthesis of purer VO_2_ powder with high crystallinity. Hua et al. employed a combination of solvent-thermal and pyrolysis processes to synthesize purer VO_2_ powder under mild air conditions. Through manual grinding and etching processes, they obtained innovative VO_2_(M) nanoparticles with high crystallinity^55^. The equations for the aforementioned processes are as follows:

5 $$N{H}_{4}V{O}_{3}+{C}_{2}{H}_{6}{O}_{2}\to {N}_{2}+{VO}({OC}{H}_{2}C{H}_{2}O)$$

6 $${VO}\left({OC}{H}_{2}C{H}_{2}O\right)+{O}_{2}\to V{O}_{2}+{H}_{2}O+C{O}_{2}$$

The preparation of VO_2_ nanoparticles is a critical step in the development of VO_2_-based thermochromic smart windows. While various methods have been explored, each with its own set of advantages and limitations, the pyrolysis process stands out for its mild reaction conditions and minimal impurity introduction, which are particularly beneficial for large-scale production.

### Fabrication of VO_2_-based thermochromic films

**Fig. 6:**
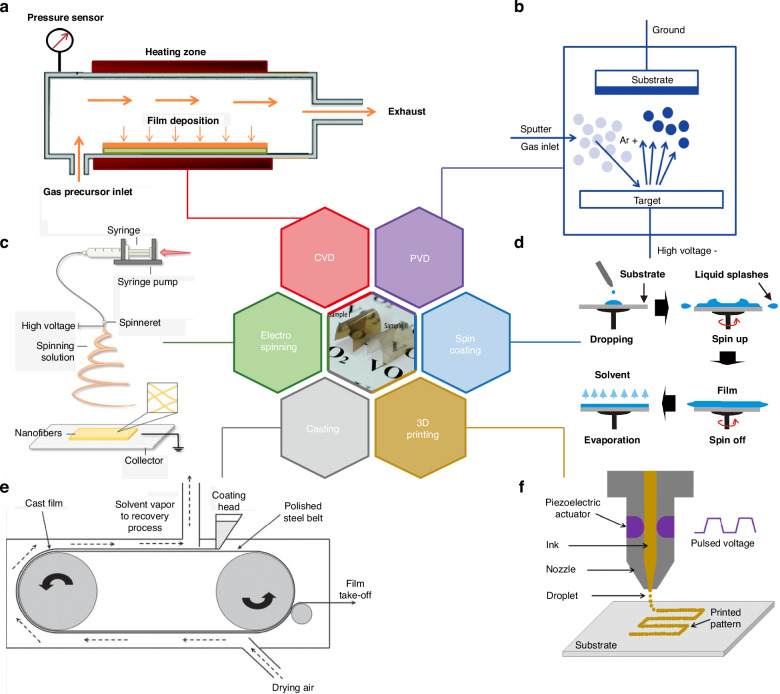
Fabrication methods for VO_2_-based thermochromic thin films. **a** Chemical vapor deposition (CVD)^174^. **b** Physical vapor deposition (PVD)^175^. **c** Electrospinning^89^. **d** Spin coating^176^. **e** Film casting^177^. **f** 3D printing^178^

Thus far, a multitude of methods have been devised to produce high-quality VO_2_ films with outstanding thermochromic properties^78–80^. These methods can be broadly categorized into chemical and physical techniques. Among them, CVD stands out as a commonly employed fabrication method for VO_2_ thin films, owing to its merits of high deposition rate, uniform structure, excellent adhesion, and scalability. In the CVD process, a precursor containing metal ions is dissolved in a solvent and then transported by an inert carrier gas to the deposition reactor, where the chemical reaction occurs, as illustrated in Fig. 6a. For instance, electric-field assisted chemical vapor deposition (EACVD)^78^ and low temperature chemical vapor deposition (LTCVD)^80^ have been developed to enhance thermochromic properties and facilitate low-cost production.

Guo et al. prepared thermochromic VO_2_ thin films (Δ*T*_sol_ 9.7% and *T*_lum_ 52.3%) by LTCVD at a lower *T*_c_. This reduction in Tc was attributed to the combined effects of strain and the presence of oxygen vacancies in the films during annealing. However, there has been limited emphasis on comparatively large-scale production, and the precursors commonly used as vanadium sources in the CVD process are typically toxic and environmentally unfriendly, such as VCl_4_^81^, VO(acac)_2_^82^, VO(OC_3_H_7_)_3_^83^, etc. PVD is an extensively researched technique for depositing high-quality VO_2_ thin films, particularly for applications like smart windows, due to its ability to produce highly homogeneous products, good repeatability, and potential for large-scale production. Examples include magnetron sputtering (MS)^63,84^ and pulsed laser deposition (PLD)^85^, etc.

As illustrated in Fig. 6b, MS is a vacuum process used to deposit thin films on substrates by applying a high voltage across a low-pressure gas (usually argon) to create a “plasma”. Energized plasma ions strike the target and cause atoms from that target to be ejected with enough energy to travel to and bond with the substrate. While PVD methods offer superior quality and crystallinity in the fabrication of VO_2_ thin films compared to other techniques, the complexity and expense of the equipment, along with high maintenance costs, severely limit their practical widespread application in smart windows^86^. Furthermore, persisting issues include poor antioxidizability and weatherability^87^. Hence, there is a demand for facile, low-cost, eco-friendly, scalable, and high-quality fabrication methods for VO_2_-based thin films.

Electrospinning is a technique wherein a high electric field is directly applied to obtain fibers with diameters ranging from nanometers to micrometers^88^. This process typically comprises four components: a high-voltage power supply, a syringe pump, a spinneret, and a collector, as illustrated in Fig. 6c^89^. It is commonly employed in the fabrication of organic-inorganic film materials^90^.

In comparison with existing methods, the electrospinning technique is straightforward, mature, versatile, economical, and efficient for preparing continuous nanofibers with high porosity. These fibers have been proven to possess exceptional optical properties suitable for radiative-cooling applications^91^. Li et al. utilized electrospinning to prepare W-doped VO_2_/PVP composite fiber mats and identified two modes of the multiple scattering-absorbing process when light was absorbed by the fiber mats^92^. Due to the constitution of the fibers in the electrospun film, internal scattering is one of the reasons of low *T*_lum_ for electrospun thermochromic films. Therefore, proper *T*_lum_-enhanced techniques are necessary. Lu et al. firstly fabricated PMMA-VO_2_ transparent composite smart film (Δ*T*_sol_ 6.9% and *T*_lum_ 21%) with antioxidizability and super-hydrophobicity by electrospinning method, attributed to the gathered VO_2_ embedded in the fiber and aligned along the fiber axis. Subsequent heat treatment process significantly enhanced its *T*_lum_^87^.

Spin coating is a solution-based technique employed to deposit uniform thin films onto flat substrates. Typically, a small amount of coating material is applied to the center of the substrate, which is then rotated at high speed to spread the coating material through centrifugal force, as illustrated in Fig. 6d. Zhao et al. synthesized W-VO_2_ thermochromic films (Δ*T*_sol_ 8.8% and *T*_lum_ 41.5%) on an amorphous glass substrate using a simple spin coating method, which is economical, straightforward, and practical. However, high-speed spinning becomes challenging as the substrate size increases, making film thinning difficult. Additionally, the material efficiency of spin coating is relatively low^93^, which limits its application for large-scale production.

In film casting, a solution (referred to as “dope”) of a polymer in a solvent is prepared and then applied to a moving substrate belt with a smooth surface^94^. The wet film levels on the belt before entering the drying zone, where the solvent is completely removed to form a solid layer. Subsequently, the dry film is peeled from the belt, which then cycles back to the coating station, as depicted in Fig. 6e.

Gao et al. were the first to prepare transparent and flexible VSQ composite foils using an all-solution casting process. These foils exhibited a UV-shielding property and excellent thermochromic properties (Δ*T*_sol_ 15.5% and *T*_lum_ 41%)^95^. In addition to wet coating with a solution, hot-melt casting is another method utilized for film deposition. A formulation is cast into a film from either hot melt or solution without solvent. The casting process offers several advantages over other methods, including simplicity of mold, low cost, absence of residual stresses, and suitability for low-production applications. It provides superior optical characteristics with a high degree of flatness compared to flat-sheet extrusion. However, casted parts may exhibit significant shrinkage post-solidification.

3D printing, or additive manufacturing, is a process for creating three-dimensional solid objects from a digital file. It encompasses various techniques, such as direct ink writing (DIW), stereolithography (SLA), selective laser melting (SLM)/selective laser sintering (SLS), and inject printing, among others. For example, one method involves selectively depositing droplets of printing materials to form the desired structure, utilizing an X-Y-Z three-axis motion platform, spray heads, and auxiliary curing devices^96,97^, as depicted in Fig. 6f. During the printing process, the fluid is ejected from the nozzle. With the movement of the platform, the ejected droplets are accurately deposited on the printing platform, and then solidified and formed at room temperature and atmospheric pressure. This method is low-cost, fast, efficient, flexible and suitable for the roll-to-roll process of complex structures, especially for materials that are difficult to form continuous wires.

However, there are limitations in printing height and mechanical properties of products. Ji et al. reported a straightforward large-scale fabrication of uniform VO_2_ thermochromic films using the inkjet printing technique (Δ*T*_sol_: 32.4%). Large area VO_2_ films (560 cm^2^) could be obtained with excellent uniformity, with the difference in NIR transmittance of regions on the film being within 0.3%^98^.

The fabrication of VO_2_-based thermochromic films is a multifaceted process with a variety of techniques offering different benefits. CVD and PVD are recognized for their high-quality film production, yet their scalability and environmental impact present challenges. Electrospinning and spin coating provide more accessible and cost-effective alternatives, with the potential for large-scale production.

Despite the success of existing fabrication methods at the laboratory scale, challenges remain in scaling up production and ensuring environmental adaptability. Future research is recommended to focus on the development of scalable, environmentally friendly, and cost-effective fabrication techniques. Additionally, it should explore new materials and structural designs to further enhance the performance and stability of VO_2_-based thermochromic films.

## Other chromogenic materials

Despite significant efforts to enhance the optical responses of VO_2_-based thermochromic films, the overall *T*_lum_ and Δ*T*_sol_ remain constrained due to the intrinsic absorption of VO_2_. In the following section, we delve into a variety of alternative chromogenic materials that exhibit significant optical property changes, which are extensively utilized to modulate solar radiation. The potential for synergistic integration of these materials with VO_2_ will be explored, with the aim of further refining spectral transmittance. Prominent among these are electro-, gaso-, mechano-, photochromic materials, each of which is activated by specific stimuli such as an electric field, gas exchange, mechanical stress, and light exposure. These smart chromogenic technologies each bring a unique set of advantages and challenges, and they are the focus of continuous innovation to improve their functionality and efficiency. The characteristics of various chromogenic materials are summarized in Table 4.

**Table 4:** Characteristics of different chromogenic materials

Chromogenic material type	Characteristics	Fabrication methods	Cost	Pros	Cons	Ref.
Electrochromic (TiO_2_, etc.)	Reversibly changing optical properties under electric field	Electrochemical deposition, solution immersion, etc.	Variable	Easy operation, IoT integration, adjustable color, excellent performance in reversible color change	Poor UV durability for polymer-based EC materials, low coloration efficiency, cycling stability issues, indoor-use restriction	^101^
Gasochromic (WO_3_, etc.)	Displaying color change in response to chemical change or reaction	Sol-gel method, chemical vapor deposition, etc.	Low	Depth and switching speed controllable by film thickness and gas concentration, easy configuration and cost efficiency, high light transmittance	Requiring hydrogen and oxygen providing system	^105,106^
Mechanochromic (PVA, PDMS, DABBF, etc.)	Responding to mechanical stimuli and changes in surface morphologies and internal structures	Mechanical processing, embossing, etc.	Low to moderate	Simple, eco-friendly, responsive	Biaxial strain and area expansion from stretching, short fatigue life of cracks under high strength mechanical loading cycles	^109^
Photochromic (fulgide, diarylethene, spiropyran, TiO_2_, etc.)	Reversible transformation when exposed to specific wavelengths of light	Solution processing, electrochemical polymerization, etc.	Variable	Structural diversity, photochromic reversibility, processibility	Ineffective initial color restoration under UV illumination, limited photochromic library	^121^
Thermochromic (VO_2_, perovskites, hydrogels, etc.)	Changing optical properties based on temperature	physical vapor deposition, chemical vapor deposition, sol-gel method, etc.	Low	Suitable for passive light modulation	Requirements for specific spectral characteristics	^48^

**Fig. 7:**
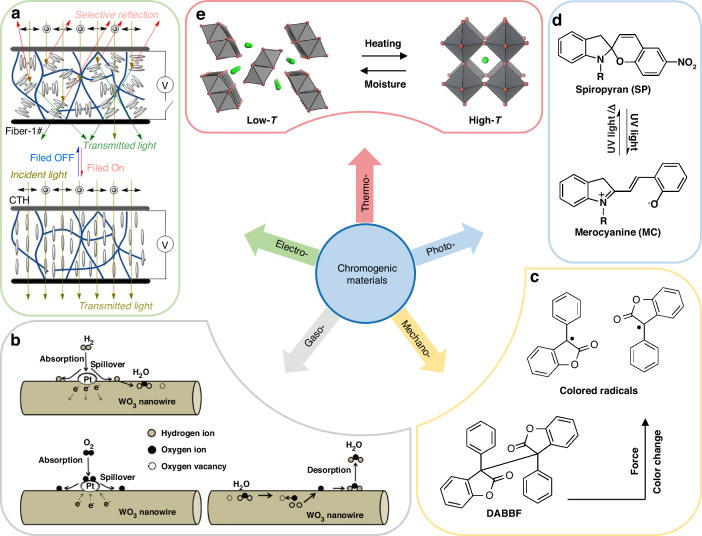
Schematic illustrations of chromogenic materials undergoing various stimuli. **a** Liquid-crystal-clad fibers by applying or removing a voltage at 25 °C^179^. **b** The adsorption/desorption of water molecules induced WO_3_ gasochromism^180^. **c** Mechanically triggered establishment of equilibrium between pale yellow DABBF and blue radicals^181^. **d** Reversible structural transformations of spiropyran under UV/visible light irradiation^116^. **e** Phase transitions of perovskites by heating or moisture^7^. Figures reproduced with permission from: (**c**), K. et al.^116^, American Chemical Society

Electrochromic (EC) materials could reversibly change the optical properties under the influence of electric field, including inorganic metal oxides (TiO_2_^99^, etc.), organic polymers (propylenedioxythiophene^100^, etc.), inorganic-organic hybrids: coordination complex^101^, prussian blue^102^, liquid crystal (Fig. 7a)^8^, etc. EC applications are composed of EC material, electrolyte, ion storage layer, etc. and sandwiched by two transparent conductive electrodes^103^. The advantages of electrochromic (EC) materials include easy operation through a control system and integration into the Internet of Things (IoT). However, challenges such as poor UV durability (especially for polymer-based EC materials), coloration efficiency, cycling stability, and indoor-use restrictions remain to be addressed in the future. Sang et al. proposed a smart window (Δ*T*_sol_: 32.4% and *T*_lum_: 0.4–40%) with a dual-band modulation function based on a simple three-layer structure of liquid crystal, VO_2_ and Al-doped ZnO, which can modulate the visible light actively by an external electric field and NIR light passively by temperature^104^.

Gasochromic (GC) materials are a type of thermochromics displaying a color change responding to a chemical change or reaction^105,106^. WO_3_ is a well-known example of GC materials. In a double-glazed structure, the internal surfaces coated with a WO3 film exhibit reversible light transmittance switching when alternately exposed to diluted hydrogen and oxygen gases, as illustrated in Fig. 7b. One advantage of GC materials, such as WO_3_, is that the depth and switching speed of color change can be controlled by adjusting the film thickness and the concentration of hydrogen and oxygen gases. Due to their straightforward and cost-effective layer configuration, as well as high light transmittance, WO_3_-based GC devices have garnered significant attention since the beginning of the 21st century^107,108^. However, a major limitation of these devices is the requirement for a system to provide hydrogen and oxygen gases.

Mechanochromic (MC) materials respond to mechanical stimuli which can deform and reconstruct the surface morphologies or internal structures with a change of their optical properties^109^, like polyvinyl alcohol (PVA)/PDMS^110^, diarylbibenzofuranone (DABBF) (Fig. 7c), etc. The MC applications are facile, eco-friendly, and responsive. The main obstacles to their commercialization include the significant applied biaxial strain and area expansion resulting from stretching, as well as the improvement of the fatigue life of cracks under high strength mechanical loading cycles without incurring catastrophic structural damage^111,112^. Ke et al. presented a bio-inspired thermo-mechanochromic dual-mode VO_2_/PVA/PDMS window (Δ*T*_sol_: 11.5% and *T*_lum_: 17–60%), to respectively control the scattering for privacy and the absorbance for energy-efficiency, which are attributed to the controllable broadband diffraction via the dynamic wrinkles and VO_2_ NPs based on LSPR^113^.

Photochromic (PC) materials undergo reversible transformation when exposed to specific wavelengths of light, which could be roughly divided into organic and inorganic PC materials. Common examples include fulgide^114^, diarylethene^115^, spiropyran^116^ (as depicted in Fig. 7d), TiO_2_^117^, etc. Compared to inorganic counterparts, organic PC materials possess great advantage in structural diversity, photochromic reversibility, and processibility^118,119^. However, simply removing the UV is not sufficient to restore most organic PC materials to their initial color (except for naphthopyran)^119,120^, and more basic research is needed to enrich the library of photochromism. Sang et al. fabricated a novel VO_2_/spiropyran composite film (Δ*T*_sol_ 23.6% and *T*_lum_ 31.55–48.6%), which could simultaneously modulate UV, visible, and NIR light with obvious color change from yellow to pink reversibly^121^.

Thermochromic materials enable a change in optical properties based on the temperature^122^, which is suitable for passive light modulation. In addition to VO_2_^48^, perovskites (Fig. 7e)^123^ and hydrogels^51^ are also very representative. They have their own spectral characteristics towards application scenarios in demand.

The exploration of alternative chromogenic materials offers a promising avenue for addressing the limitations of VO_2_-based thermochromic coatings. These aforementioned materials demonstrate significant changes in optical properties and are widely utilized in solar radiation modulation. Integrating these materials with VO_2_ has the potential to further enhance functionality in smart windows. Each of these chromogenic technologies presents unique advantages and challenges, spurring continuous innovation to enhance their efficiency and performance. Through synergistic integration, these materials can enable smart windows to achieve improved optical responses and multifunctionality, thus facilitating their broader adoption in energy-efficient building applications.

## Conclusion and outlook

Here, we present a comprehensive review on the VO_2_-based thermochromic coatings for smart windows. With the inspiring MIT transition, VO_2_ serves as a promising material for spectral transmittance modulation. Given the limitations of pristine VO_2_, such as its high transition temperature, low luminous transmittance, and weak solar-energy modulation ability, a variety of research approaches have been proposed, spanning from the crystal level to the structural level, including elemental doping and micro-nano structural engineering. Based on the latest researches, we discuss the principles and results of micro/nano-engineering in detail by categories including hybridization, core-shell structures and multilayer film design. Moreover, we summarize the fabrication methods of VO_2_ NPs and VO_2_-based thermochromic films. We also provide discussions on other chromogenic materials, which can be integrated for thermochromic smart windows as an alternative strategy to surpass the intrinsic limitation of VO_2_. Besides the above conclusions and summarizations, outlooks and prospects are discussed as follows, which aim to inspire more innovative progress and accelerate the development of this research field from the lab to the industry.

VO_2_-based thermochromic smart windows represent an important technology for increasing indoor comfort and reducing energy consumption in the building. Hence, this hotspot is promising and competitive. Some problems still need to be addressed to further widen their practical applications.

- Idealizing the optical responses including *T*_lum_, Δ*T*_sol_ and Δ*T*_NIR_. To facilitate the commercialization of VO_2_-based radiative cooling coatings, ideally Δ*T*_sol_ and Δ*T*_NIR_ should be respectively increased to 57% and 100% with high *T*_lum_. This is where researchers had made efforts upon micro/nano-engineering. Further investigations on micro/nano-engineering should be revealed with more complex core-shell micro/nanostructures, higher order optical resonances and combining with other chromogenic materials discussed in Other Chromogenic Materials.
- Continuous and various color changes. Color preference is tightly related to local customs with high versatility. For instance, a colorless coating or coloration with light blue/green is acceptable in China^124^. While gold or violet is more attractive in some areas of Southeastern Asia or Arabia^125^. More sophisticated core-shell or multilayer structure design can help us to achieve this goal.
- Stability and lifetime. Vanadium is multivalent transition element, which reveals +3, +4, +5, and mixed valences. VO_2_(M) tends to be oxidized to V_2_O_5_ or their hydroxides, especially in a humid environment. This instability leads to low lifetime in practical applications. Therefore, the antioxidizability and hydrophobicity of VO_2_-based thermochromic films are worthy of our attention. VO_2_-based core-shell micro/nano-structure design might already enhance the antioxidizability to some extent, while affect the luminous transmittance of thermochromic films. Introducing hydrophobic groups on the surface of films to trap water molecules by grafting, etc. chemical modification means^126^ and protective film fabrication methods, i.e., electrospinning^127^ would be bright solutions.
- Superior fabrication techniques for VO_2_-based thermochromic coatings need to be of large-scale, low-cost, high-quality, high efficiency, and eco-friendly, etc.
- Broadening the multifunctionality of smart windows, e.g., introducing flexibility and foldability, integration of solar cells for electricity generation, integrated display for interactivity, etc.
- Exploiting VO_2_ in advanced applications for radiative cooling^128^, warming^129^, and camouflage^130,131^: The potential of VO_2_ extends beyond smart windows into the realms of radiative cooling, warming, and camouflage technologies. The thermochromic nature of VO_2_ allows for the development of coatings that can dynamically regulate heat exchange with the environment, making them ideal candidates for applications where temperature control and thermal management are critical. For radiative cooling, VO_2_ coatings could automatically adjust their thermal emissivity to reflect more solar energy and emit more thermal radiation, thereby reducing the need for artificial cooling systems. In the context of warming, VO_2_ could be engineered to absorb more solar energy while reducing thermal radiation loss, providing an energy-efficient approach to heating. Moreover, in the field of camouflage, VO_2_-based coatings could change their thermal signature in response to environmental conditions, making objects less detectable by infrared sensors. The challenge lies in tailoring the phase transition temperature of VO_2_ to operate effectively under varying conditions and integrating these coatings into practical systems while maintaining their optical and thermal performance. Future research should focus on the scalable production of VO_2_ coatings with tailored properties, their integration with other advanced materials for enhanced performance, and the development of dynamic control systems for responsive thermal management.

**Fig. 8:**
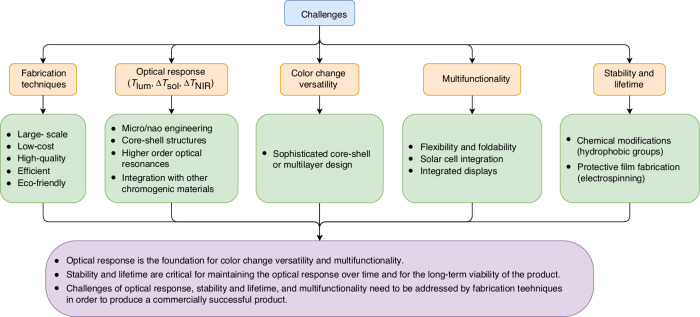
Challenges in the development of VO_2_-based thermochromic smart windows

To further elucidate the challenges and corresponding solution paths in the development of VO_2_-based thermochromic smart windows, we have prepared a summary, as depicted in Fig. 8. This figure provides readers with a clear visualization of the strategies proposed to address the challenges identified in this review, facilitating a deeper understanding of the research landscape and potential avenues for future exploration.
